# Eye size recognition of self and others among people with self-face dissatisfaction

**DOI:** 10.1177/20416695221148039

**Published:** 2023-02-03

**Authors:** Izumi Ayase, Masaki Mori, Takaaki Kato

**Affiliations:** Graduate School of Media and Governance, 119863 Keio University, Japan; Faculty of Environment and Information Studies, 12869 Keio University, Japan; 119863 Faculty of Environment and Information Studies, Keio University, Japan

**Keywords:** face dissatisfaction, face recognition, self-face, other-face, perceived eye size

## Abstract

Previous studies have shown that individuals visually recognize their eye size as larger than the actual. However, it is unclear whether this cognitive tendency occurs in people with high self-face dissatisfaction. Therefore, this study aimed to investigate whether the cognitive size of one's own and others’ eyes differs according to the degree of self-face dissatisfaction. Participants comprised 32 college students (5 males, 27 females; age: 21.3 ± 2.11) who completed the Face Dissatisfaction Scale (FDS) and a face recognition memory task. The task was to choose whether their or their friends’ eyes in the face photos with changed eye size were larger or smaller than their actual eye size. The cognitively equivalent eye size to the actual one was estimated from a psychophysical function. We conducted a correlation analysis of the total scores on the FDS and the point of subjective equality (PSE) of eye size. We found a high negative correlation between the FDS and the PSE of own eye size. There was also a high positive correlation between the FDS and the PSE for all others’ faces. Thus, high self-face dissatisfaction is differentially associated with cognitive distortions of the face, depending on whether it is self or other.

It is generally believed that people can recognize themselves in face photos. This mechanism of self-face recognition is demonstrated when one visually distinguishes between everyday objects and the faces of others. It is known that the self-face is perceived and processed in preference to non-self-related information because it is closely related to oneself. Previous studies have shown that self-relevant information is considered more likely to be encoded and retrieved because it is seen and heard often in daily life (Bola et al., 2021; Burton et al., 2005; Żochowska et al., 2021). Keenan et al. (1999) conducted a three-choice reaction time task in which participants’ dominant (right) and nondominant (left) hands were used depending on whether the presented stimulus was a self-face, familiar face, or unfamiliar face. The results showed that the nondominant hands’ reaction time was shorter when the stimulus was a self-face rather than a familiar or unfamiliar face. This result suggests that the self-face is processed faster in the right hemisphere than the other-face. Faster recognition of the self-face was also shown when the self-face and the other-face photos were used as target stimuli in a visual search task. Devue et al. (2009) used a visual search task to show that it took longer to detect the target stimulus when the presented stimulus (either the interfering or target stimulus) contained both the self-face and others’ face photos than when it contained only others’ face photos. This suggests that the self-face is more likely to receive attention and be processed preferentially to other faces. Previous studies have shown that the self-face captures more attention than not only the faces of others with low familiarity but also the faces of others with high familiarity, such as celebrities and close friends, by using cognitive experiments (inverted face effect: Keenan et al., 1999; Keyes & Brady, 2010; selective attention: Bola et al., 2021; visual search: Devue et al., 2009) and brain activity measurement (event-related potentials: Alzueta et al., 2019). Thus, the self-face is perceptually and cognitively processed as different information from other visual stimuli.

People may apply makeup, adjust their eyebrows and hair, or undergo surgical and dermatological procedures to make their self-face more attractive. Previous studies have found that large eyes and a symmetrical face are more likely to be perceived as attractive (Cunningham, 1986; Geldart et al., 1999; Jones et al., 2007; Little et al., 2007; Rhodes et al., 1998). Faces can be manipulated in certain ways using makeup or image processing techniques, so that the size of the eyes or the texture of the skin is perceived as different from the person's real face (Jones et al., 2018; Kobayashi et al., 2017; Morikawa et al., 2015; Takehara et al., 2021). Matsushita et al. (2015), Morikawa et al. (2015), and Muto et al. (2019) examined the extent to which larger eyes are perceived when eye makeup is applied to the upper eyelid. The participants’ task in these studies was to simultaneously observe a standard face photo and a comparison stimulus and adjust the comparison stimulus to be the same size as the standard stimulus. The standard stimulus was a face photo with eye shadow and mascara. The comparison stimulus was a face photo in which the eye size was modified between approximately 90% and 110% of the standard stimulus without eye makeup. The eye size of the comparison stimulus perceived to be equivalent to the standard stimulus was estimated. The comparison stimulus was estimated to be equal to the standard stimulus with 105% eye size. This shows that the eye makeup effectively alters the perceived eye to approximately 5% larger than the actual size. Since larger eyes are likely to be seen as attractive by others (Cunningham, 1986; Geldart et al., 1999), eye makeup enhances perceived attractiveness.

The act of trying to make oneself look beautiful suggests the desire to have a more attractive face than one's own. Excessive desire and obsession with one's own facial attractiveness can cause psychological distress and interfere with daily activity. The condition in which negative thoughts and feelings toward one's own physical characteristics occur is called body dissatisfaction (Ambo et al., 2012). When body dissatisfaction is strongly triggered, resulting in repetitive checking behavior and avoidance of social situations, it may be a diagnosis of body dysmorphic disorder (American Psychiatric Association, 2022). Various psychological scales are used to measure body dissatisfaction. For example, the Body Image Concern Inventory can measure physical disfigurement concerns, one of the symptoms of body dysmorphic disorder, and has been developed and widely used in various countries around the world (Littleton & Breitkopf, 2008; Littleton et al., 2005; Luca et al., 2011; Tanaka et al., 2011). In Japan, in recent years, it has been reported that people with body dysmorphic disorder are particularly unsatisfied with one's own face (Nabeta, 2011; Nagata et al., 2005). Japanese who perform surgical cosmetic procedures have been reported to perform approximately 92% of their procedures on the face and head (International Society of Aesthetic Plastic Surgery, 2019a, 2019b). Dissatisfaction with one's own face is a particularly important psychological characteristic of body dissatisfaction. Ayase et al. (2022) have developed a new psychometric scale to assess the dissatisfaction with self-face. This scale is named the “Face Dissatisfaction Scale” (hereinafter referred to as the “FDS”). The FDS consists of three factors: avoidance factor, fear factor, and obsessive thinking factor, and its reliability and validity have been confirmed in approximately 1,000 general people aged 17 to 42 years. Furthermore, the FDS uses data from 25 people with body dysmorphic disorder and 106 college students, and a cutoff point of 122 points is calculated based on the signal detection theory (Ayase et al., 2021). The FDS is a psychometric scale that can measure individual differences in dissatisfaction with one's face and is expected to play a role as a screening test to identify the pathophysiology of body dysmorphic disorder.

Self-face perception is known to differ depending on an individual's psychological characteristics. Many previous studies have reported that patients with body dysmorphic disorder differ from general participants in their perception of self-face (Feusner et al., 2010; Grocholewski et al., 2012; Yaryura-Tobias et al., 2002). Buhlmann et al. (2008) examined whether body dysmorphic disorder affects the perceived attractiveness of other people's faces as well as one's own face. A total of 19 participants with body dysmorphic disorder and 21 general participants were asked to rate attractiveness using a seven-point Likert scale for 12 photos of others’ faces with high attractiveness, 12 with average attractiveness, 12 with low attractiveness, and one photo of their self-face. The results showed that people with body dysmorphic disorders were more likely to rate the attractiveness of their own faces lower than that of others and healthy people. Furthermore, participants with body dysmorphic disorder rated their own faces as equally attractive as other average attractive face photos, whereas general participants rated their own faces as more attractive than other average attractive face photos. These results may mean that the perceived attractiveness of one's own and others’ faces depends on the level of dissatisfaction with one's own body. One of the reasons that body dysmorphic disorder sufferers estimate low levels of self-face attractiveness may be because they strongly dislike for or are dissatisfied with the minor flaws or internal features of their own bodies. What kind of cognitive processing mechanism causes such negative self-perception?

One way to identify this mechanism is to examine how dissatisfaction with one's own face affects memory representations of one's own and others’ faces. That is, it is necessary to clarify the differences between actual and represented faces to investigate the cognitive distortion of the self-face. Wen and Kawabata (2014) presented participants in an experiment with several face photos with enlarged and reduced eye size and asked them to select the face photo that was perceived to be closest to the actual image. The faces used in the experiment were the participants’ own faces and the faces of others who were familiar to the participants. As a result, it can be seen from the data that the participants selected the face photo with eyes of the same size in reality (i.e., the size of the eyes was not manipulated) as the person in the photo regardless of whether it was the face of another person or the participant's own face. However, a higher percentage of respondents selected the face photo with larger-than-real-size eyes as the person in the photo for the self-face than for the other-face. This result suggests that the self-face and the other-face have different memory representations. Although previous studies have concluded that the general tendency is for the self-face to be perceived as more attractive than it really is (Epley & Whitchurch, 2008; Hine & Okubo, 2021), it is unclear what kind of distortions occur in mental representations among those who are dissatisfied with their self-face.

This study aimed to determine how individuals who are dissatisfied with the self-face retain memory representations of the faces of people they know. The present study included the FDS, a psychological scale measuring dissatisfaction with the self-face, and a face recognition memory task that measured memory representations of eye size for the self-face and the faces of other people known to the participants. This is because the eyes are one of the essential parts that form the impression of the face (Jones et al., 2018). Eyes can be evaluated for perceived size using a psychophysical method, and cosmetic techniques to make them appear larger have been studied (Matsushita et al., 2015; Morikawa et al., 2015; Muto et al., 2019). In general, the larger the eyes, the higher the facial attractiveness (Cunningham, 1986; Geldart et al., 1999). Based on this, we may assume that the greater the dissatisfaction with one's own face, the higher the tendency to perceive one's face as less attractive; consequently, the greater the dissatisfaction with one's face, the smaller one's own eyes are perceived to be in contrast to their actual size. Accordingly, those who are highly dissatisfied with their own face may remember others’ eyes as being larger than they actually are.

## Method

### Participants

The participants were 32 university students (5 males, 27 females; age 21.3 ± 2.11 years). The participants performed a face recognition memory task based on memories of their own faces and the faces of people they were familiar with in their daily lives. The participants provided oral and written informed consent prior to participating in the study. Before starting the experiment, the participants were informed of the content of the study and scope of use of the face photos. They agreed to have their own face photos taken and manipulated. This study was conducted with the approval of the Keio University SFC Research Ethics Committee. The approval number was 369.

### Stimulus and Apparatus

The visual stimuli used in the face recognition memory task were images of the participants’ own faces and the faces of people who were familiar to them. The participants and known persons belonged to the same community and saw each other once a week. Known persons were selected from two communities. Participants who routinely wore makeup were instructed to wear makeup as usual. Those who did not usually wear makeup were required not to wear makeup.

The participants’ faces were photographed using a single-lens reflex camera (Nikon D5600 and AF-S DXNIKKOR 18-140mm f/3.5-5.6 G ED VR; 35mm equivalent focal length was 24 mm), seated with their backs to a uniform gray paper (Superior, BPS-1305; No. 4) and fixed at a 60 cm shooting distance. When photographed, the participants were required to have a neutral facial expression with their eyes open. Faces were illuminated at 612 lx using three LED fluorescent lamps (Ecolica Inc., ECL-LD4EGD-L3ANN; correlated color temperature 6500 K) for color evaluation in a room with no light from outside.

**Figure 1.**
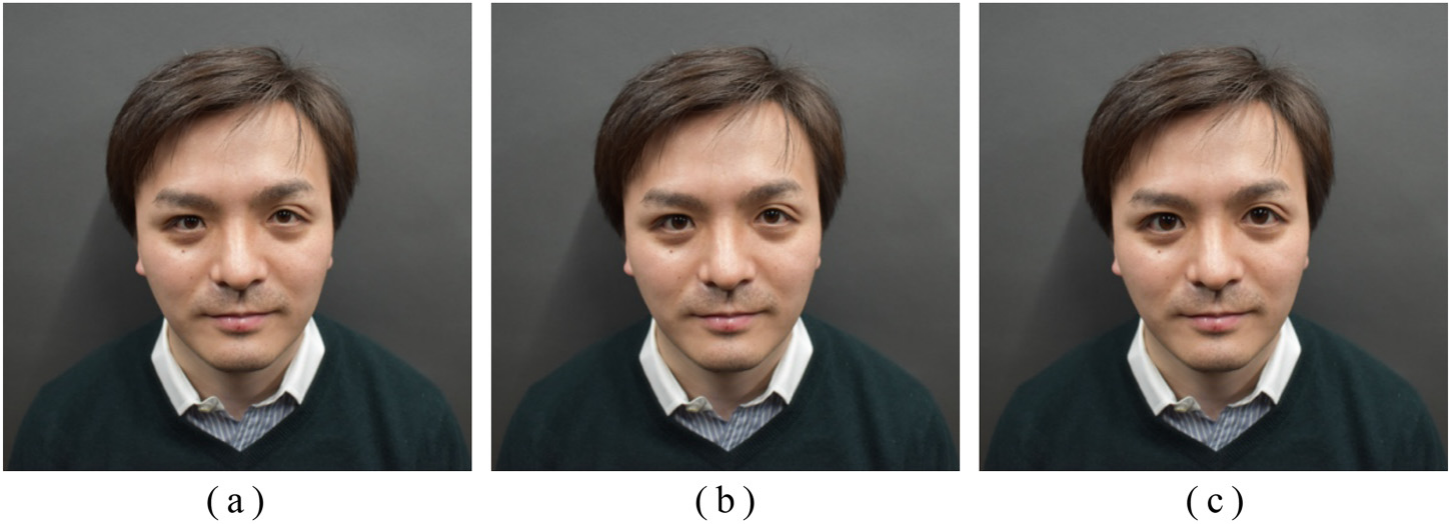
Examples of stimuli. The eye size was (a) modified by 20% smaller, (b) unmodified, and (c) modified by 20% larger.

Figure 1 shows an example of a face stimulus with manipulated eye size. The eye size in face photos was scaled using the Eye Size slider of Adobe Photoshop CS5.1 (Adobe Inc.) by approximately ± 5%, ± 10%, ± 15%, or ± 20% in each length and width. The sizes of the sclera, iris, and pupil were scaled according to the exposed portion of the eye size manipulated by the Eye Size slider. No other facial features (e.g., face height, width, skin tone) were processed in this experiment. Therefore, the nine-face photos were created per person, including the unprocessed face photos. All of the face photos were presented life-size on a calibrated monitor (EIZO Corporation, ColorEdge CS2420-Z, 24.1 inch, 1920 × 1200 pixels resolution) in a dark room.

### Procedure

Participants responded to the FDS and performed a face recognition memory task.

The FDS is created and tested for reliability and validity by Ayase et al. (2022). It assesses avoidance, fear, and obsessive thinking about the self-face appearance. Participants rated the degree to which the 27 items applied to them on a seven-point Likert scale (1 = absolutely inappropriate, 2 = inappropriate, 3 = slightly inappropriate, 4 = neutral, 5 = slightly appropriate, 6 = appropriate, and 7 = absolutely appropriate). The higher the total score of the FDS, the higher the dissatisfaction with one's own face. The cutoff for body dysmorphic disorder was 122 points (Ayase et al., 2021).

For the face recognition memory task, participants observed a life-size face photo presented for 500 ms on a monitor and were asked whether the eyes of the person in the photo were larger or smaller than that person's real eyes. This psychophysical task was constant method performed 720 times by each participant in that there were 20 repetitive trials for four blocks of face photos (one of oneself and three of other people), with each block containing the nine-face photos with different eye sizes. Other-face A was one of the two male photos (A1 or A2) known to the participant, other-face B was one of the two female photos (B1 or B2) known to one of the participants, and other-face C was one female photo known to all participants. The original eye size of the male face A1 was 2.7 cm (width) × 1.2 cm (height), and that of the male face A2 was 3.1 cm × 1.3 cm. The female face B1 was 3.3 cm × 1.4 cm, and the female face B2 was 2.4 cm × 1.1 cm. The female face C was 3.4 cm × 1.5 cm. Male face A (A1 or A2) and female face B (B1 or B2) were used differently in the experiment because the known faces differed depending on the community to which they belonged.

The study period was from August to October 2021. The order of presentation of the nine-face photos was counterbalanced within each block. The experiment was created using PsychoPy 3.1.5 (Peirce, 2007; Peirce et al., 2019) and controlled by a personal computer (Dell Technologies Inc., Dell G5 5500). Participants pressed the left arrow key when they perceived the eyes as smaller than the real size and the right key when they perceived them to be larger. The visual distance was 57 cm using a chin rest from a monitor. Data were analyzed using the statistical analysis software R version 4.1.2 (R Core Team, 2021).

## Results

Binary logistic regression analysis using the maximum likelihood estimation method was conducted for each participant and each person in the face photos, using the binary data obtained in the face recognition memory task as the dependent variable and the magnification of the eyes in the face photos as the independent variable. The logistic equations were fitted to estimate the point of subjective equality (hereinafter referred to as the “PSE”). The PSE is the magnification ratio of the memorized eye size to the actual eye size. The larger the PSE from zero, the larger the eyes in the face photo were perceived to be, and vice versa. The significance level was 5% in this study.

**Figure 2.**
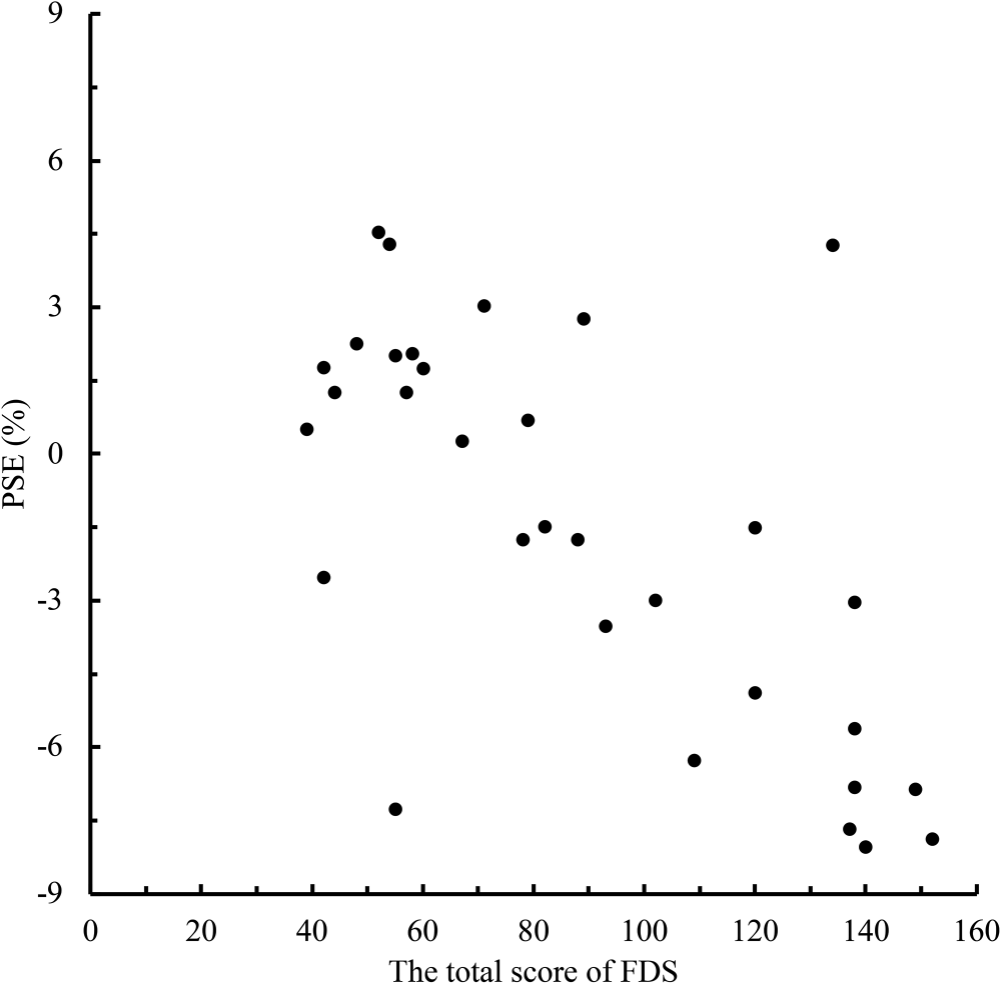
Relationship between the total scores of Face Dissatisfaction Scale (FDS) and point of subjective equality (PSE) scores in self-face condition.

**Figure 3.**
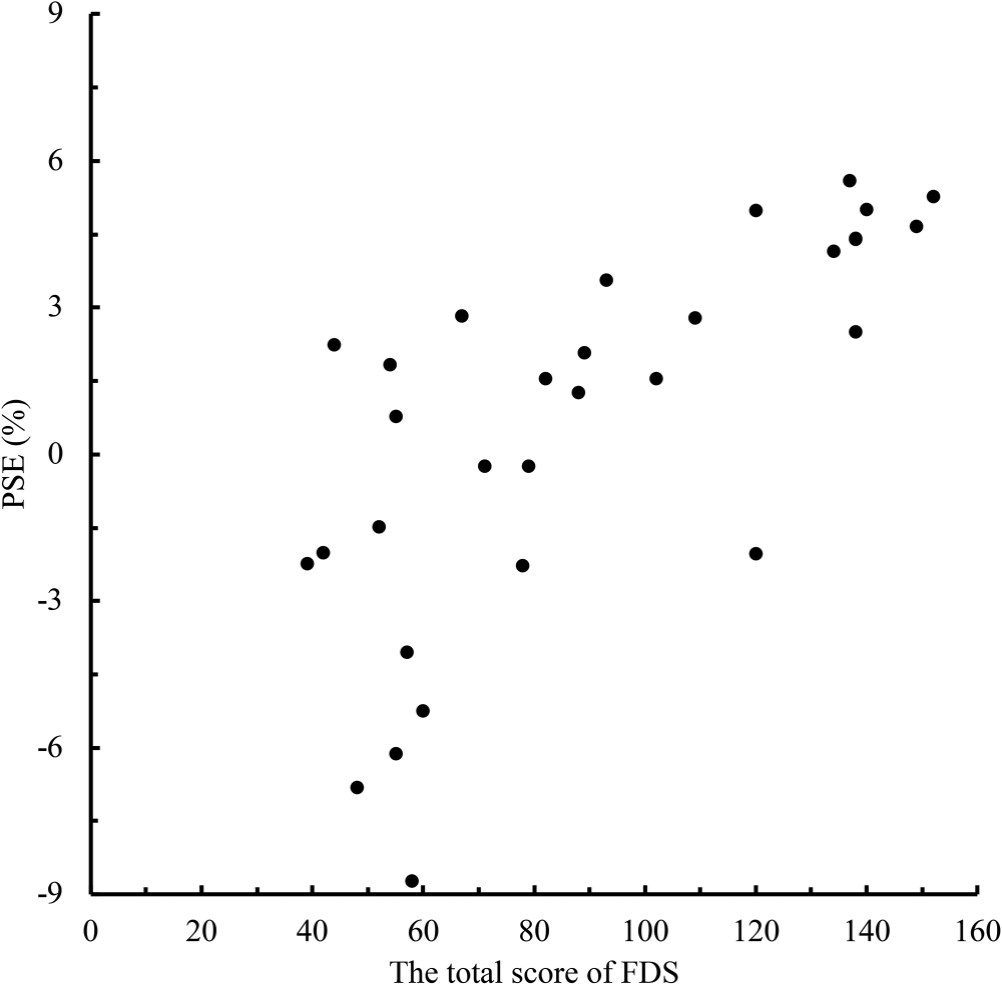
Relationship between the total scores of Face Dissatisfaction Scale (FDS) and point of subjective equality (PSE) scores in other-face A condition.

**Figure 4.**
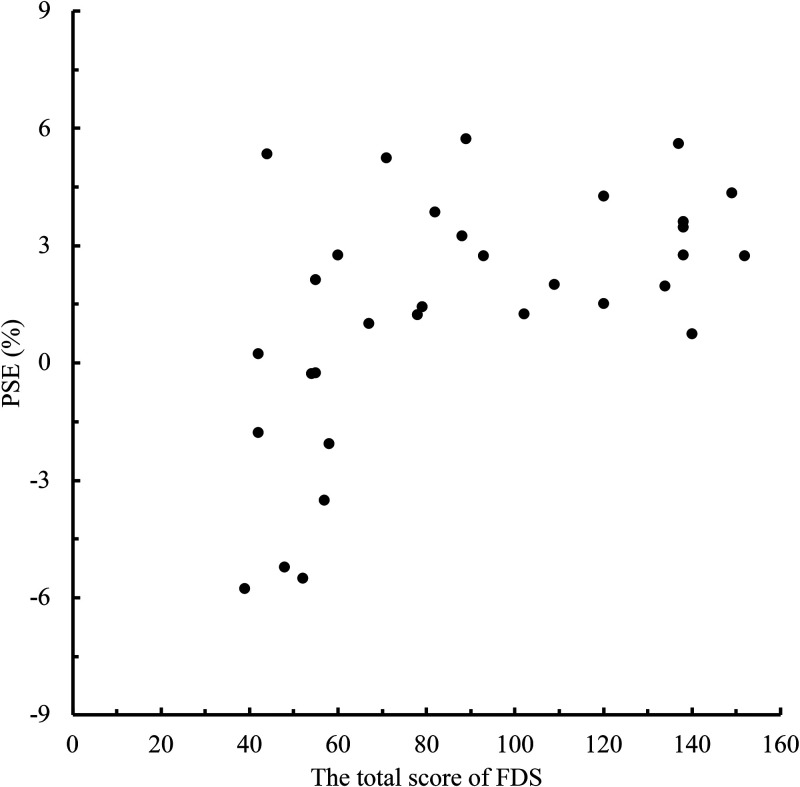
Relationship between the total scores of Face Dissatisfaction Scale (FDS) and point of subjective equality (PSE) scores in other-face B condition.

**Figure 5.**
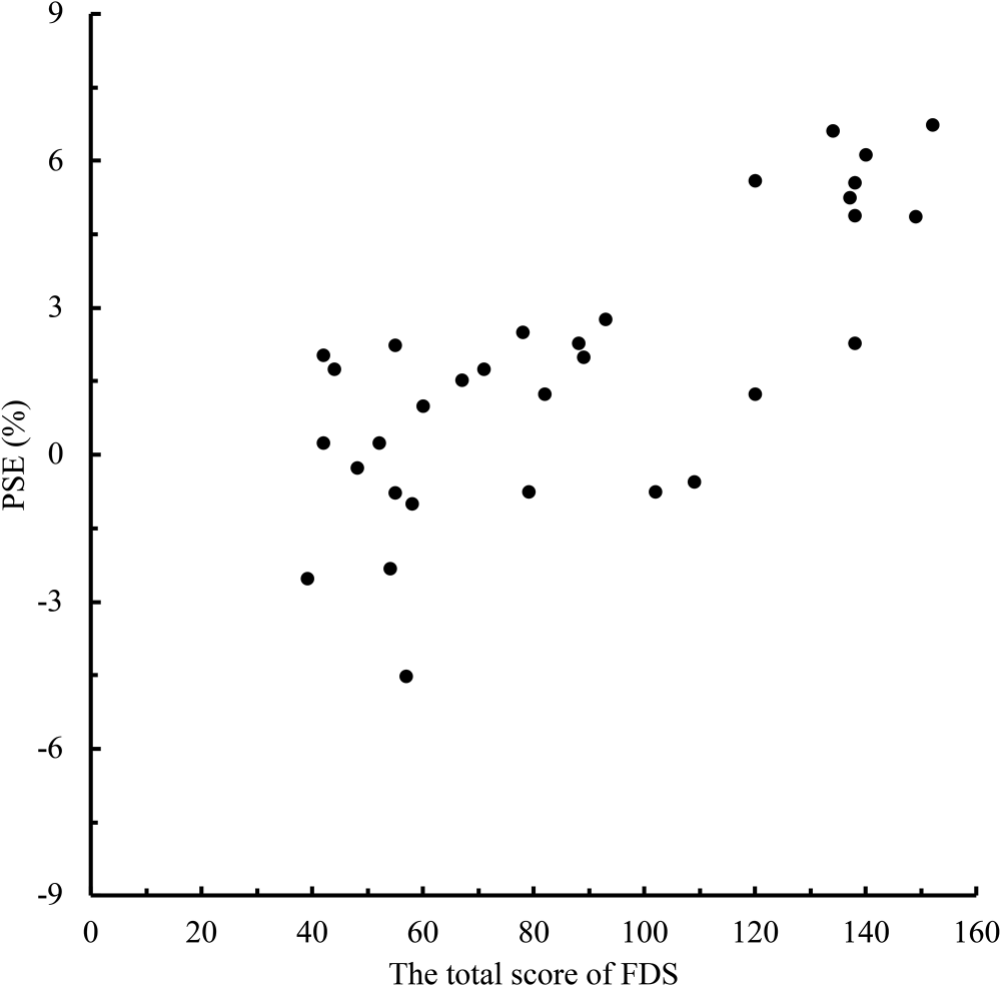
Relationship between the total scores of Face Dissatisfaction Scale (FDS) and point of subjective equality (PSE) scores in other-face C condition.

To clarify the relationship between the level of face dissatisfaction and the memory of eye size in the self-face and the other-face, we conducted a correlation analysis between the total FDS score and PSE for each person in the face photos (Figures 2–5). When the face photo was a self-face, there was a high negative correlation between FDS and PSE [*r* =  − .66, *p* <.001]. In contrast, when the face photo was the other-face there was a high positive correlation between FDS and PSE [other-face A condition: *r* = .72, *p* <.001; other-face B condition: *r* = .54, *p* <.001; other-face C condition: *r* = .75, *p* <.001]. In addition, we conducted a correlation analysis of the PSE with the total score for each of the FDS subfactors (avoidance, fear, obsessive thinking). Table 1 shows a significant negative correlation between the FDS subscales and PSE in the self-face condition and a significant positive correlation between the FDS subscales and PSE in the other-face condition.

**Figure 6.**
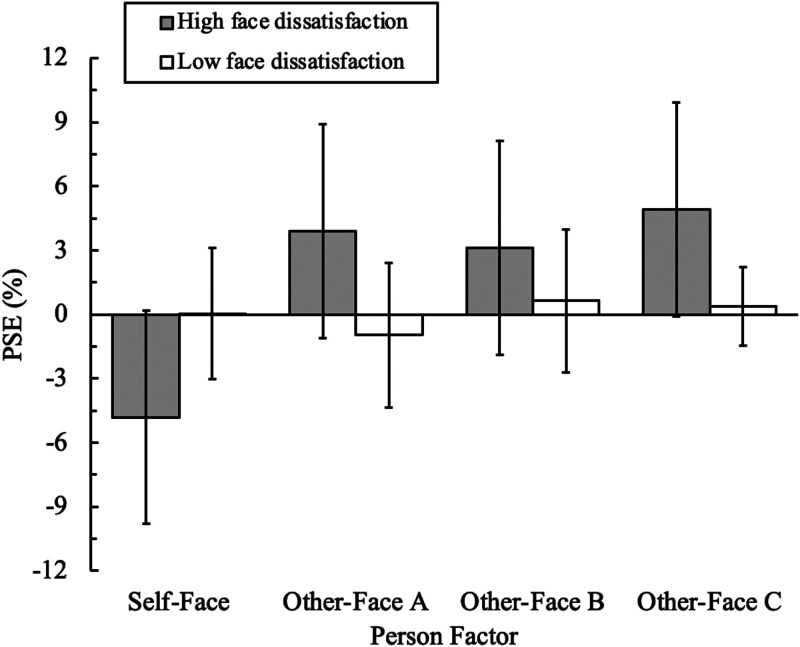
Means and standard deviations of point of subjective equality (PSE) for self-faces and other-faces each of low and high groups of face dissatisfaction. The PSE is the magnification ratio of the perceived eye size to the actual eye size.

**Table 1.** Correlation matrix between the subscale of FDS and PSE in each condition.

	FDS		
	Avoidance	Fear	Obsessive thinking
Self-face	−.67 ***	−.62 ***	− .59 ***
Other-face A	.68 ***	.68 ***	.72 ***
Other-face B	.53 ***	.46 ***	.56 ***
Other-face C	.74 ***	.67 ***	.73 ***

*** *p* <.001.

FDS= Face Dissatisfaction Scale; PSE = Point of Subjective Equality.

The 32 participants were classified into two groups: a high face dissatisfaction group (M = 136.60, SD = 9.83) with 10 participants scoring higher than the FDS cutoff of 122 points, and a low face dissatisfaction group (M = 66.54, SD = 20.11) with 22 participants scoring lower than the cutoff.

Figure 6 shows the means and standard deviations of the PSE for each group for the self-face and the other-face. A two-way analysis of variance was conducted using face dissatisfaction (high and low groups) and person (self-face, other-face A, other-face B, and other-face C) as factors for the PSE scores, with only the person factor having a response. The results showed that the main effect of the person factor and the interaction between the face dissatisfaction and the person factors were significant [*F*(3, 90) = 17.84, *p* <.001, *η_p_^2^*  = .37; *F*(3, 90) = 18.08, *p* <.001, *η_p_^2^*  = .37, respectively]. The main effect of the face dissatisfaction factor was not significant [*F*(1, 30) = 8.26, *ns, η_p_^2^*  = .21]. Since the interaction was significant, a simple main effect test was conducted. The results showed that the simple main effects of the face dissatisfaction factor were significantly different in the self-face, other-face A, and other-face C conditions [*F*(1, 90) = 20.69, *p* <.001, *F*(1, 90) = 20.78, *p* <.001; *F*(1, 90) = 18.26, *p* <.001, respectively] and not significantly different in the other-face B condition [*F*(1, 90) = 5.40, *ns*]. The simple main effect of the person factor was significantly different in the high face dissatisfaction group condition [*F*(3, 27) = 30.85, *p* <.001] and not significantly different in the low face dissatisfaction group condition [*F*(3, 63) = 1.29, *ns*]. To clarify the differences between conditions in the person factor, we conducted multiple comparisons using the Bonferroni method (using the approximately 0.083% each of test for an overall significance level at 5%). The results showed that there were significant differences between the self-face and the other-face A conditions, between the self-face and the other-face B conditions, and between the self-face and the other-face C conditions [all, *p* <.05]. There were no significant differences among the other-face A, other-face B, and other-face C conditions.

To clarify whether the memory of eye size in the person in the face photo differed from the real one depending on the level of face dissatisfaction, a one-sample t-test was conducted on the PSE (self-face: M = 0.03, SD = 3.07; other-face A: M = −0.96, SD = 3.39; other-face B: M = 0.64, SD = 3.34; other-face C: M = 0.37, SD = 1.85). The results showed that in the high face dissatisfaction group, the PSE for the self-face condition was significantly smaller than 0, and that for the other face condition was significantly larger than 0 [self-face condition: *t*(9) =  − 3.9, *p* <.01; other-face A condition: *t*(9) = 5.47, *p* <.001; other face-B condition: *t*(9) = 6.75, *p* <.001; other-face C condition: *t*(9) = 8.66, *p* <.001]. In the low face dissatisfaction group, the PSEs for all conditions in the person factor were not significantly different from 0 [self-face condition: *t*(21) = 0.04, *ns*; other-face A condition: *t*(21) =  − 1.3, *ns*; other-face B condition: *t*(21) = 0.87, *ns*; other-face C condition: *t*(21) = 0.92, *ns*].

## Discussion

The purpose of this study was to determine whether the memory of eye size in one's own face and the faces of others differs depending on the level of dissatisfaction with one's own face. The results showed that those with high face dissatisfaction remembered their own eyes as being smaller and those of others as being larger than those with low face dissatisfaction, the latter recalling the eyes to be the same size as in reality, regardless of whether they were their own or those of others. Thus, the higher the level of face dissatisfaction, the more likely participants were to remember their own eyes as smaller and others’ eyes as larger than the actual size.

Previous studies have repeatedly reported that larger eyes tend to be perceived as more attractive (Cunningham, 1986; Geldart et al., 1999). Based on this, the present results suggest that when dissatisfaction with the face is high, the self-face is perceived as less attractive than it actually is, while the opposite is true for others’ faces. Whereas, when face dissatisfaction is low, memory representations of the face may be equivalent to the actual face regardless of it being the self-face or others’ face. Thus, people with high face dissatisfaction may underestimate the attractiveness of their own faces and overestimate the attractiveness of others’ faces compared to those with low face dissatisfaction. Buhlmann et al. (2008) has shown that people with body dysmorphic disorder tended to rate their own facial attractiveness lower than that of general people when rating the attractiveness of their own face and that of unfamiliar others. The present results support the findings of Buhlmann et al. (2008), using an eye size recognition task. However, the recognition memory task for the self-face and the other-face used in the present study did not require an explicit rating of facial attractiveness. Negative cognitive bias toward the self-face also occurs in the implicit recognition of eye size, a robust phenomenon in individuals with high dissatisfaction with their own bodies.

The present study found that the eye size of memory representations in the self-face and the other-face was equal to those of the real faces when face dissatisfaction was low. Previous studies have repeatedly reported that the remembered representations of eyes in self-faces are larger than real ones in general adolescents and adults (Epley & Whitchurch, 2008; Hine & Okubo, 2021; Wen & Kawabata, 2014). These previous studies did not consider the psychometric characteristics of the participants, and it is not clear what kind of psychometric data was collected from participants. Even though the youths and adults targeted in the previous studies are considered low face dissatisfaction or body dysmorphic disorder participants with high face dissatisfaction, the results differ from the trends obtained in the present study. One of the possible reasons for the different results is the various tasks of the psychological experiment regarding eye size perception. In the present study, a two-alternative forced-choice paradigm was used in which participants were asked to determine whether their eyes were larger or smaller than the actual size of a single presented stimulus in one trial. In a previous study, a multiple-alternative forced-choice paradigm was used, in which participants had to select one of many face photos while visually comparing and referring to them. The present study may have differed from the previous studies because the matching was conducted using only the representations remembered from past daily experiences as cues.

Previous research has shown that the faces of friends and famous people are recognized faster than those of strangers (Burton et al., 2005, 2011). The participants and the other person whose face was used as the stimuli in this study belonged to the same community and saw each other once a week. Therefore, eye size was judged in this study based on their daily memories. When judgments of eye size are based on daily memories, the results may vary depending on the relationship between people of stimuli faces and participants, such as the frequency they meet in person or the intimacy. Interestingly, the psychophysical experiments in this study were established on the basis of everyday memories, even though the standard stimuli were not presented. In addition, the present study did not refer to original facial photos in the experiments or use a phase for learning the standard eye size. Therefore, it is not clear whether the distortion of eye size perception was due to perceptual or memory bias. In order to clarify this fact, it is necessary to conduct experiments in which standard and comparison stimuli are presented simultaneously or in which the participants are asked to memorize another person's face immediately before the recognition task.

At least three limitations of this study can be cited. First, our sample consisted of a limited number of participants. It is not clear whether the high face dissatisfaction group identified by the FDS can be considered as an equal sample to psychiatrically diagnosed body dysmorphic disorder patients. Body dysmorphic disorder patients are known to be dissatisfied not only with their eyes but also with their skin, nose, and body shape (International Society of Aesthetic Plastic Surgery, 2019a, 2019b). This study dealt with only a small subset of symptoms of body dysmorphic disorder. Second, the study data were obtained during the COVID-19 pandemic, a period in which many people wear hygiene masks daily to prevent the spread of COVID-19 infection (Chen et al., 2020; Ministry of Health, Labour and Welfare, 2022), and their mouths are covered by masks in face-to-face situations, except for classes and meetings in an online environment. Masks do not cover the eyes, but it is not certain how they affect the formation of memory representations of faces. It has also been reported that the attractiveness of other people's faces is higher when they are not wearing masks than when they are (Miyazaki & Kawahara, 2016). It has been reported that during the COVID-19 pandemic, the attractiveness and health of a person wearing a mask is perceived as higher than before the pandemic (Kamatani et al., 2021). The results of this study suggest that the habit of wearing a mask in response to the spread of COVID-19 may have had some effect on face perception. Third, this study measured dissatisfaction but not satisfaction with the self-face. It has been reported that perception and cognition are affected by positive evaluations and attitudes toward the self, such as potential self-esteem (Koole & DeHart, 2007). To clarify individual differences in the perceived eye size of self and others by considering positive evaluations of the self-face, it is necessary to develop a psychometric tool that assesses satisfaction with one's face, such as a “Face Satisfaction Scale,” in the future.

The present cross-sectional survey revealed the effects of face dissatisfaction on distortions in the perception of the self-face and the other-face. This study needs to clarify whether face dissatisfaction increases or decreases over time with any intervention. We discuss two ways to reduce face dissatisfaction. The first way is a makeup intervention. Morikawa et al. (2015) reported several conditions for eye makeup to be perceived larger. It has also been reported that larger eyes are more likely to be perceived attractively by others than smaller eyes (Cunningham, 1986; Geldart et al., 1999). Does makeup alter eye size perception and facial attractiveness regardless of facial dissatisfaction? If makeup can make eyes perceived by others as larger than they are, will those with high levels of face dissatisfaction perceive their own eyes as larger? The second way is a psychotherapeutic intervention. Cognitive-behavioral therapy alleviates symptoms of individuals with body dysmorphic disorder (Harrison et al., 2016). Previous studies have measured the efficacy of cognitive-behavioral therapy using the Yale-Brown Obsessive-Compulsive Scale Modified for Body Dysmorphic Disorder (Phillips et al., 1997) and the Body Dysmorphic Disorder Examination (Rosen & Reiter, 1996). It needs to be clarified what cognitive mechanisms are responsible for symptom relief. The present study investigated some aspects of face cognition characteristics in individuals with high face dissatisfaction through a cognitive experiment on eye size. Unlike psychometric scales used in previous studies, the experimental tasks in the present study may be able to measure the degree of cognitive distortion directly. A longitudinal survey combining this study's method with cognitive-behavioral therapy has excellent potential to help us understand how intervention methods affect cognitive aspects and by what mechanisms face dissatisfaction can be reduced.
